# Comparative Transcriptomic Analysis Reveals the Negative Response Mechanism of Peanut Root Morphology and Nitrate Assimilation to Nitrogen Deficiency

**DOI:** 10.3390/plants12040732

**Published:** 2023-02-07

**Authors:** Lijie Li, Xiangguo Cheng, Xiangjun Kong, Peipei Jia, Xiaohui Wang, Lei Zhang, Xiaotian Zhang, Yi Zhang, Zhiyong Zhang, Baohong Zhang

**Affiliations:** 1 Henan Collaborative Innovation Center of Modern Biological Breeding and School of Life Science and Technology, Henan Institute of Science and Technology, Xinxiang 453003, China; 2 Institute of Crop Sciences, Chinese Academy of Agricultural Sciences, Beijing 100081, China; 3 Department of Biology, East Carolina University, Greenville, NC 27858, USA

**Keywords:** peanut (*Arachis hypogaea* L.), nitrogen deficiency, roots morphology, transcriptome

## Abstract

Root architecture plays a fundamental role in crop yield, which is sensitive to nitrogen fertilizer. Although it is well studied that nitrogen fertilizer significantly promotes peanut (*Arachis hypogaea* L.) growth and yield, less information was available on how its root development responds to nitrogen deficiency. In this study, the growth and development of roots were inhibited, as indicated by the significantly decreased root dry weight and length and the lateral root number, especially under 10 days of nitrogen deficiency treatment. The activities and the expression of the genes related to nitrogen assimilation enzymes including nitrate reductase, glutamine synthetase, glutamate dehydrogenase, and glutamine oxoglutarate aminotransferase and the genes encoding the nitrate transporters were significantly decreased under 10 days of nitrogen deficiency treatment, which may lead to a decrease in nitrate content, as indicated by the significantly decreased nitrogen balance index. Transcriptome sequencing revealed a total of 293 (119 up- and 174 downregulated) and 2271 (1165 up- and 1106 downregulated) differentially expressed genes (DEGs) identified after five and ten days of nitrogen deficiency treatments, respectively. Bioinformatic analysis showed that these DEGs were mainly involved in nitrate transportation and assimilation, phytohormone signal transduction, and the lignin biosynthesis pathway. Furthermore, a putative schematic diagram of nitrogen deficiency inhibiting root growth was established, which gives us a better understanding of nitrogen metabolism in peanut roots and a theoretical basis for improving nitrogen use efficiency.

## 1. Introduction

Nitrogen is one of the essential macronutrients that not only limits plant growth and yield but also has an important impact on the environment [1]. Since chemical nitrogen fertilizer significantly improved crop yield, it has been extensively used in agricultural production [2]. However, the overuse of nitrogen fertilizer brings a series of problems, including soil acidification and eutrophication [3] and the suppression of agricultural sustainable development [4]. Therefore, it is urgent to explore a strategy that reduces the investment in chemical nitrogen fertilizer without decreasing crop yields.

Nitrate (NO_3_^−^) is the main absorbing form of inorganic nitrogen fertilizer [5], which can be acquired from the soil by plants through the operation of a large number of nitrate transporters (NRTs) [1]. After root uptake of NO_3_^−^, NO_3_^−^ can be directly metabolized and stored in the root vacuole, but a larger part of NO_3_^−^ is transported to the aboveground shoot. Nitrate is first assimilated by nitrate reductase (NR) in the cytoplasm, and its product NO_2_^−^ is then transported to chloroplasts/plastids, where it is converted to NH_4_^+^ by nitrite reductase (NiR) [6]. The generated NH_4_^+^ is then assimilated into organic nitrogen via glutamine synthetase (GS)/glutamate synthase, also called the glutamine oxoglutarate aminotransferase (GOGAT) cycle pathway or the glutamate dehydrogenase (GDH) pathway, of which the GS/GOGAT pathway is the main pathway in the NH_4_^+^ assimilation pathway [6]. Nitrogen deficiency and/or irrational application of nitrogen fertilizers lead to unbalanced N accumulation and distribution in plants through nitrogen uptake and transport systems [1], which in turn leads to abnormal levels of nitrogen assimilation and metabolism [7], ultimately leading to a decrease in biomass and yield [8].

It has been documented that NO_3_^−^ not only provides structural material for plant growth and development but also works as a vital signal molecule participating in many plant life activities including regulating root system architecture (RSA) [1]. In this process, NRT1.1 is particularly important because of its ability to transport auxin, which has been shown to regulate the occurrence and elongation of lateral roots [9,10]. RSA represents the spatial arrangement of root and its morphology, functions to anchor plants, acquires water and nutrients, and is a fundamental aspect of crop productivity [11]. The plasticity of root development is critical for optimizing the capture of NO_3_^−^ in continuously changing and NO_3_^−^-deficient soil environments [12]. Studies showed that root development presented a diverse phenotype while suffering from different degrees of nitrogen deficiency [1]. Moderate concentrations of nitrate have a stimulating effect on primary and lateral root growth, whereas a severe lack of NO_3_^−^ supply inhibits the formation and elongation of lateral root [13,14]. For instance, low nitrogen availability increased the root length in rapeseed [15] while inhibiting root elongation in wheat [16] and peanut [17]. Therefore, it can be predicted that there may be different signaling pathways regulating the growth and development of plant roots under NO_3_^−^ deficiency conditions [1]. Exploring the molecular mechanisms of plant root development under nitrogen deficiency could provide a theoretical basis for enhancing nitrogen use efficiency and reducing nitrogen fertilization. To the best of our knowledge, several critical genes involved in root development under nitrogen deficiency have been identified using plant mutants, such as NRT1.1 [18] and AFB3 [19] in *Arabidopsis* and Aux/IAA in maize [20]. Although mutants can help understand the molecular mechanism of nitrogen metabolism in roots to a certain degree, it is difficult to understand the genome-wide regulation of nitrogen on root development and response to nitrogen metabolism. With the quick development of high-throughput deep sequencing RNA-seq, transcriptome analysis has become a powerful tool to illuminate the molecular mechanism of plant morphology and physiology. For instance, RNA-seq has been employed to study RSA in wheat [16] and sorghum [21] under nitrogen deficiency. However, limited studies have been reported on the molecular mechanism of peanut root response to nitrogen deficiency.

Peanut (*Arachis hypogaea* L.) is a worldwide-cultivated economic crop due to its high-quality oil and protein. During peanut cultivation, approximately 68–116 kg of nitrogen ha^−1^ was fixed by rhizobium [22]. It was considered that sufficient nitrogen can be supplied for peanut growth and development by rhizobium symbiotic nitrogen fixation (SNF), almost eliminating the demand for nitrogen fertilizer. However, subsequent research pointed out that nitrogen fertilizer significantly improved peanut pod yields even though peanut was inoculated [23], indicating that SNF could not satisfy the nitrogen fertilizer demand of high peanut yields. Therefore, nitrogen fertilizer has been extensively used to improve peanut yield. Unfortunately, excessive nitrogen fertilizer input leads to reduced production [24]. Thus, the regulation of nitrogen fertilizer application is still a critical step in peanut productivity.

In previous experiments, we found that the peanut variety “Yuhua 37” is sensitive to nitrogen deficiency treatment and that its root growth was inhibited when cultured in nutrient solution containing 0.1 mM NO_3_^−^, and we further found that it was closely related to the regulation of miRNAs [17], but the relationship between the inhibition of root growth caused by nitrogen deficiency and mRNA expression was unknown. In this study, we systematically investigated the effect of nitrogen deficiency on peanut root development at the morphological, biochemical (enzymatic), and molecular levels. Comparative transcriptomic analysis was conducted at different stages (before and post phenotypic change) to explore the crucial genes and metabolism pathways that respond to nitrogen deficiency in peanut. The tested hypothesis was that nitrogen deficiency would influence gene expression and further manipulate peanut root development as well as nitrate assimilation. This study allows us a better understanding of nitrogen metabolism in peanut roots and provides a theoretical basis for improving nitrogen use efficiency.

## 2. Results

### 2.1. Nitrogen Deficiency Affected the Morphology of Peanut Roots

**Figure 1:**
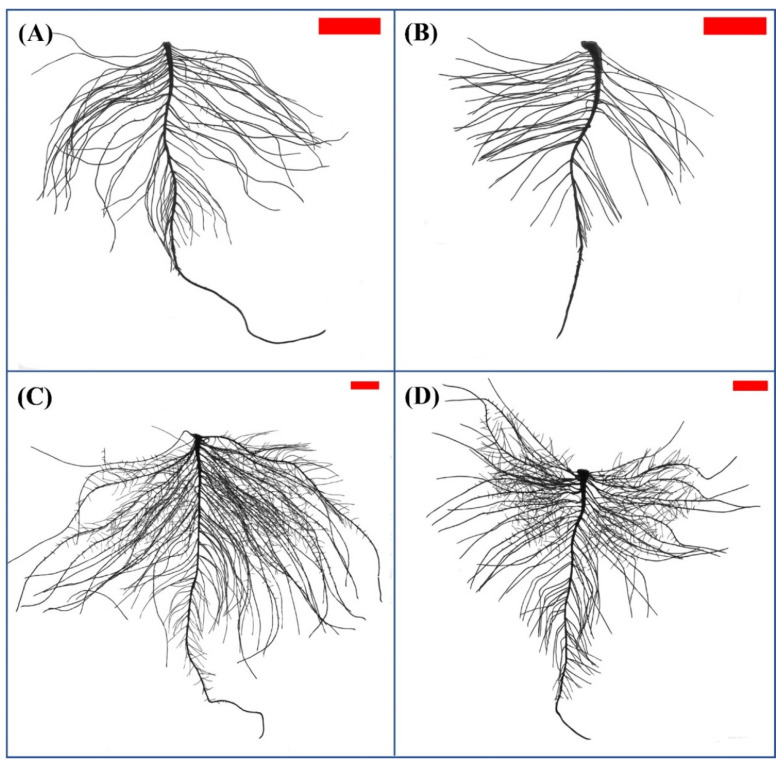
Effect of nitrogen deficiency on peanut root development and growth. Root morphology of peanut under high nitrogen (**A**) and low nitrogen (**B**) for five days and that under high nitrogen (**C**) and low nitrogen (**D**) for ten days. The original size of the target bar is 1 cm high and 2 cm wide.

Peanut root development was significantly inhibited by nitrogen deficiency after ten days of treatment, while the morphology of roots was similar between the two treatments after five days (Figure 1A–D). Compared with HN treatment, the RDW, TRL, TRSA, TRV, NLR, LRD, and PRL were decreased by 26.1%, 30.4%, 31.9%, 33.3%, 17.3%, 29.7%, and 34.4% under ten days of LN treatment (Table 1), respectively. These results indicated that LN hampered peanut root development by suppressing the elongation and proliferation of lateral roots.

### 2.2. Nitrogen Deficiency Affected the Nitrogen Content of Peanut Leaves

**Figure 2:**
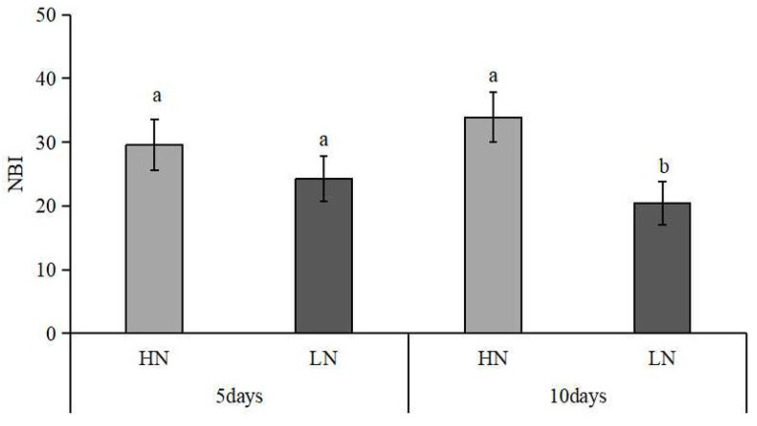
Effect of nitrogen deficiency on the leaf nitrogen balance index (NBI). HN: high nitrogen; LN: low nitrogen. The different letters on the bars indicate a significant difference at *p* < 0.05 between the high-nitrogen and low-nitrogen treatments on the same day.

Under the condition of nitrogen deficiency, the NBI value of leaves was decreased gradually with the extension of stress time, and the difference between the HN treatment and LN treatment reached a significant level at 10 days of nitrogen deficiency treatments (Figure 2). Compared with the HN treatment, the value of NBI was decreased by 39.68% (*p* < 0.05) after ten days of nitrogen deficiency treatments.

### 2.3. Nitrogen Deficiency Affected the Activities of Nitrate Assimilation Enzymes in Peanut Roots

**Figure 3:**
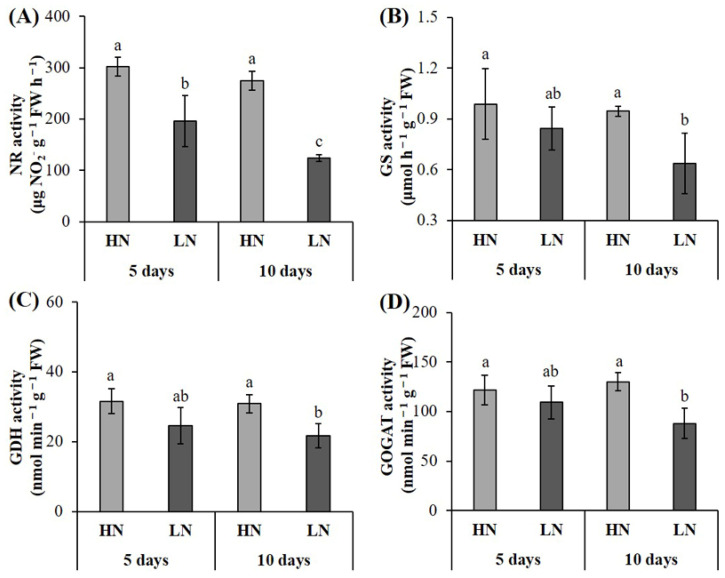
Effect of nitrogen deficiency on enzyme activities in peanut roots. Nitrate reductase (NR) (**A**), glutamine synthetase (GS) (**B**), glutamate dehydrogenase (GDH) (**C**), and glutamine oxoglutarate aminotransferase (GOGAT) (**D**). HN: high nitrogen; LN: low nitrogen. The different letters on the bars indicate a significant difference at *p* < 0.05 between the high-nitrogen and low-nitrogen treatments on the same day.

Nitrogen deficiency significantly inhibited the activities of NR activity in peanut roots (Figure 1A). Compared with the HN treatment, the NR activity decreased by 35.01% and 54.93% (*p* < 0.05), respectively, after five and ten days of LN treatments. For GS, GDH, and GOGAT, their activities were significantly decreased under 10 days of nitrogen deficiency treatments; however, there was no significant impact observed between HN and LN at five days of treatment (Figure 3B–D). Compared with the HN treatment, the activities of GS, GDH, and GOGAT decreased by 32.63%, 29.45%, and 32.43% (*p* < 0.05), respectively, after ten days of LN treatment.

### 2.4. Nitrogen Deficiency Induced Aberrant Expression of Genes

RNA-seq was employed to identify genes associated with peanut response to nitrogen deficiency and study the underlying molecular mechanism. After sequencing, a total of 82.2 Gb of clean data was generated for 12 cDNA libraries with at least 6.51 Gb for each sample, and the Q30 of all libraries was more than 92.63%. Between 92.63 and 95.59% of sequences were mapped to the peanut reference genome, among which 75.74–81.35% were uniquely mapped (Table S2). A total of 56,058 genes were identified, including 6727 novel genes (Table S3).

**Figure 4:**
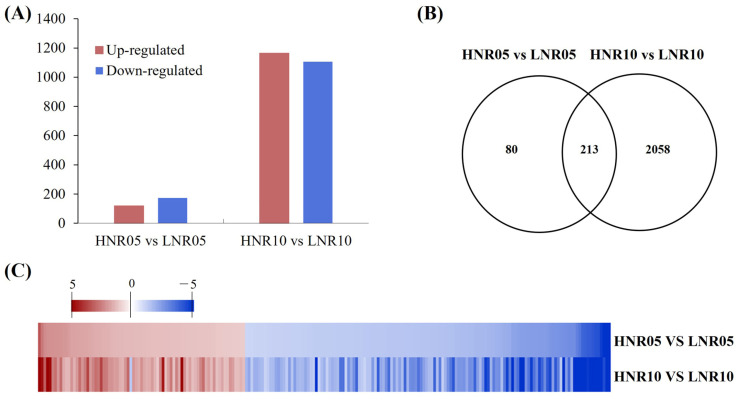
Effect of nitrogen deficiency on gene expression in peanut roots. Statistical analysis of DEGs during nitrogen deficiency treatment for 5 and 10 days. (**A**) The number of significantly upregulated and downregulated expressed genes; (**B**) Venn diagrams of DEGs from two root comparisons; (**C**) heatmap of overlapped DEGs in HNR05 vs. LNR05 and HNR10 vs. LNR10 comparisons.

By comparing different samples, a total of 293 (119 up- and 174 downregulated) and 2271 (1165 up- and 1106 downregulated) DEGs were obtained from HNR05 vs. LNR05 and HNR10 vs. LNR10, respectively (Figure 4A, Tables S4 and S5). Of them all, 213 DEGs were overlapped in HNR05 vs. LNR05 and HNR10 vs. LNR10 comparisons (Figure 4B). Interestingly, the majority of these DEGs presented similar expression trends except *arahy.Tifrunner.gnm2.ann1.6AY1PL* (*A.6AY1PL*), which was upregulated in HNR05 vs. LNR05 but downregulated in HNR10 vs. LNR10 (Figure 4C, Table S6). Therefore, we speculated that the 213 DEGs were sensitive to nitrogen deficiency and play critical roles in the nitrogen metabolism process. With the stress period increasing, more DEGs were detected under nitrogen deficiency, suggesting that nitrogen deficiency continuously leads to more physiological and metabolic changes in peanut roots.

### 2.5. GO and KEGG Annotation Analysis of DEGs

**Figure 5:**
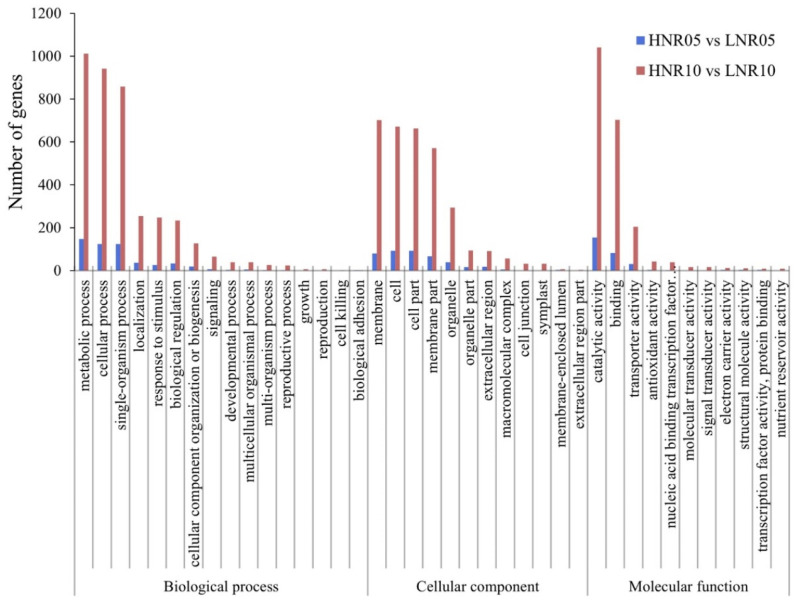
GO annotation terms of DEGs from HNR05 vs. LNR05 and HNR10 vs. LNR10.

To interpret the biological function of DEGs, GO annotation analysis was performed (Figure 5). For both stages of roots, a total of 235 and 1694 DEGs were classified into 30 and 39 terms of three GO categories: “biological process (BP)”, “cellular component (CC)” and “molecular function (MF)”, respectively. The mainly enriched GO terms in both groups were similar. For instance, DEGs in two groups were mainly divided into metabolic process, cellular process, and single-organism process in BP; membrane, cell, cell part, and membrane part in CC; and catalytic activity, binding, and transporter activity in MF. However, some unique terms were identified in HNR10 vs. LNR10 such as growth, reproduction, cell junction, symplast, and nucleic acid binding transcription factor activity. These terms might relate to the development and growth of the roots under nitrogen deficiency. Furthermore, GO significant enrichment analysis of DEGs was conducted with KS < 0.05. The detailed information is listed in Table S7.

To further identify the metabolism pathway of DEGs, KEGG pathway enrichment analysis was performed using a corrected *p* < 0.05 (Table 2). As a result, a total of five KEGG terms including starch and sucrose metabolism, pentose and glucuronate interconversions, phenylpropanoid biosynthesis, glutathione metabolism, and nitrogen metabolism were significantly enriched in HNR05 vs. LNR05. These results implied that DEGs participating in the above metabolic processes were sensitive to nitrogen deficiency in peanut roots. With the stress period increasing, more pathways were altered to root response to nitrogen deficiency in HNR10 vs. LNR10 such as cysteine and methionine metabolism, cyanoamino acid metabolism, tyrosine metabolism, phenylalanine metabolism, isoquinoline alkaloid biosynthesis, and taurine and hypotaurine metabolism.

### 2.6. DEGs Participated in Nitrate Transportation and Assimilation

**Figure 6:**
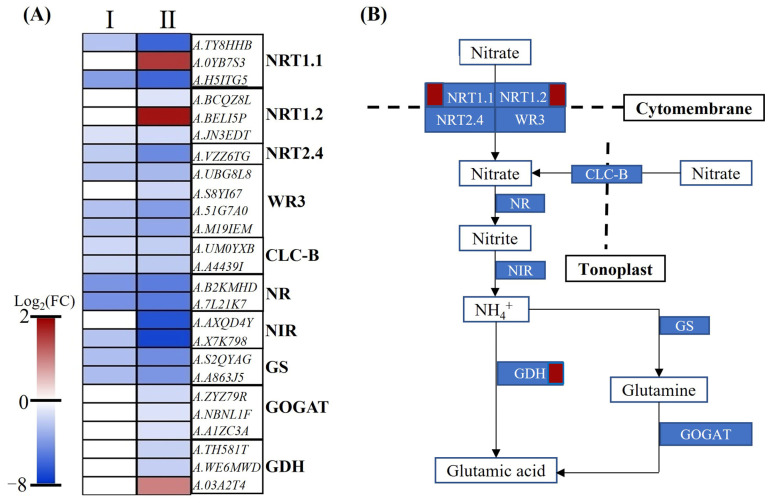
DEGs participated in nitrate transportation and assimilation. (**A**) Heatmap of DEGs related to nitrate transportation and assimilation. I: HNR05 vs. LNR05; II: HNR10 vs. LNR10. (**B**) Nitrate transportation and assimilation process in HNR10 vs. LNR10. For each rectangular shape, red shapes represent upregulated DEGs, while blue shapes represent downregulated DEGs.

Nitrate transporters play an important role in the process of nitrate uptake and transport. In this study, a total of 13 DEG-encoded nitrate transporters protein subunits (NRT1.1, NRT1.2, NRT2.4, WR3, and CLC-b) were obtained. At the initial stage of nitrogen-deficit stress, nine DEGs were identified and all of them were downregulated. With the stress period increasing, the above DEGs showed larger-fold changes, and more DEGs (13 DEGs, 11 down- and two upregulated) were detected (Figure 6A). For the nitrate assimilation process, a total of two NRs, two NIRs, two GSs, three GOGATs, and three GDH protein-encoding genes were differentially expressed in HNR10 vs. LNR10. All of them were downregulated in two comparisons except *A.03A2T4,* which was upregulated (Figure 6A,B, Table S8).

### 2.7. DEGs Related to Plant Hormone Signal Transduction

**Figure 7:**
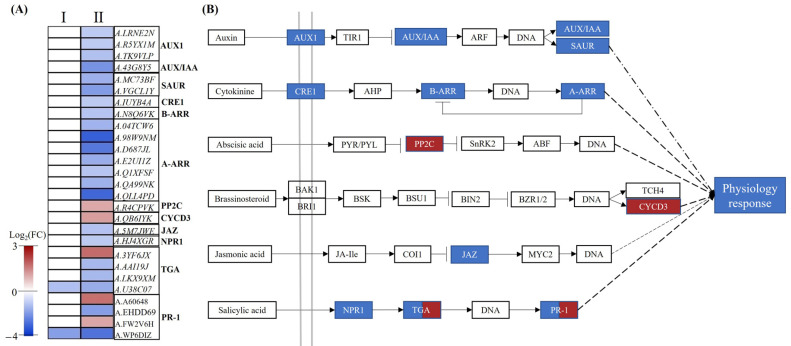
DEGs related to plant hormone signal transduction. (**A**) Heatmap of DEGs related to plant hormone signal transduction pathways. I: HNR05 vs. LNR05; II: HNR10 vs. LNR10. (**B**) Plant hormone signal transduction process in HNR10 vs. LNR10. For each rectangular shape, red shapes represent upregulated DEGs, while blue shapes represent downregulated DEGs.

Plant hormone signal transduction pathways control nitrate transport and assimilation to adapt nutrition for root growth and development. After five days of treatment with nitrogen deficiency, one DEG encoding TGA and one DEG encoding PR-1 were downregulated, while 27 DEGs were detected with ten days of treatment. All DEGs involved in auxin, cytokinin, and jasmonic acid signal transduction were downregulated, while one DEG encoding PP2C and one DEG encoding CYCD3 were upregulated, which are involved in abscisic acid and brassinosteroid signal transduction pathways, respectively. Nine DEGs were detected in salicylic acid transduction (Figure 7A,B, Table S9).

### 2.8. DEGs Involved in Lignin Biosynthetic Pathway

**Figure 8:**
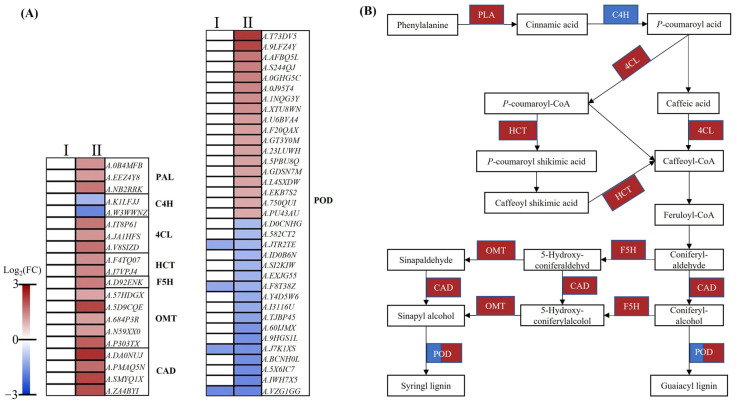
DEGs participated in lignin biosynthetic pathway. (**A**) Heatmap of DGs related to lignin biosynthetic pathway. I: HNR05 vs. LNR05; II: HNR10 vs. LNR10; (**B**) lignin biosynthetic pathway in HNR10 vs. LNR10. For each rectangular shape, red shapes represent upregulated DEGs, while blue shapes represent downregulated DEGs.

Peanuts, as a dicotyledonous plant, mainly produce G- and S- lignin through the phenylpropanoid biosynthesis metabolism pathway. In this study, a total of 55 DEGs participated in root lignin biosynthesis under nitrogen deficiency, which encode the subunits phenylalanine ammonia-lyase (PAL), cinnamate-4-monooxygenase (C4H), 4-coumarate--CoA ligase (4CL), hydroxycinnamoyl-type acyl-transferase (HCT), ferulic acid 5-hydroxylase (F5H), flavone 3′-O-methyltransferase (OMT), cinnamyl alcohol dehydrogenase (CAD), and peroxidase (POD) (Figure 8A,B, Table S10). For HNR05 vs. LNR05, four DEGs encoding PODs were detected and all of them were downregulated. In terms of HNR10 vs. LNR10, there were 2 and 17 downregulated DEGs encoding C4H and POD, respectively. Thus, it could be inferred that these downregulated DEGs may be related to root growth inhibition under nitrogen deficiency. Additionally, more than half of DEGs were POD protein-encoding genes, indicating that POD is a critical enzyme in the lignin synthesis process.

### 2.9. DEGs Encoded Transcription Factors (TFs)

A total of 17 (2 upregulated and 15 downregulated) and 185 differentially expressed TFs (86 upregulated and 99 downregulated) were identified in HNR05 vs. LNR05 and HNR10 vs. LNR10, respectively (Table S11). These TFs belonged to several transcription factor families, including WRKY (WRKY proteins), NACs (NAM/ATAF/CUC), MYB (v-myb avian myeloblastosis viral oncogene homolog), lateral organ boundaries (LOB), and C2H2 (C2H2 and C2HC zinc fingers superfamily protein) families.

### 2.10. Putative Interaction Networks of Identified DEGs

**Figure 9:**
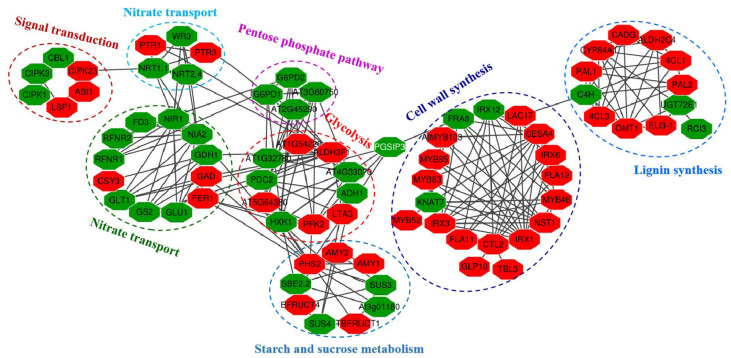
Predicted protein–protein interaction networks of all DEPs in roots under 10 days of nitrogen deficiency. Red hexagons represent upregulated DEPs; green hexagons represent downregulated DEPs. The full names of all the abbreviations above are shown in Table S12.

**Figure 10:**
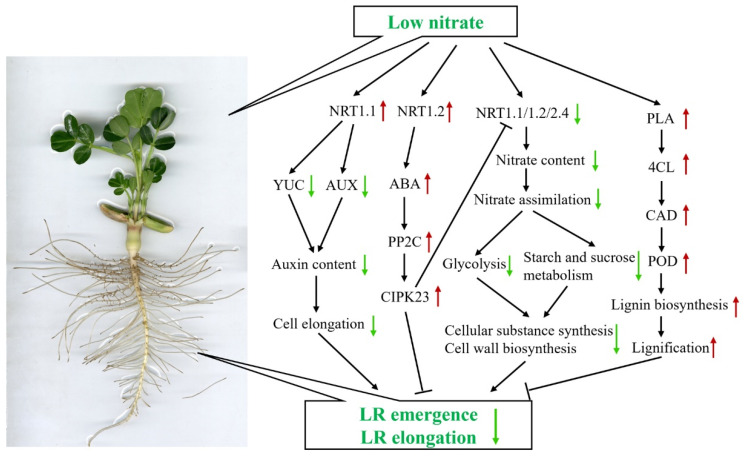
A proposed model for the mechanism underlying the inhibition of peanut roots by nitrogen deficiency. Notes: Red arrows represent upregulated and green arrows represent downregulated genes. ABA: abscisic acid; CAD: Cinnamyl alcohol dehydrogenase; 4CL: 4-coumarate--CoA ligase; NRT1.1: nitrate transporter 1.1; NRT1.2: nitrate transporter1.2; PLA: phenylalanine ammonia-lyase; POD: peroxidase; PP2C: phosphatase 2C.

All amino acid sequences of DEGs (HNR10 vs. LNR10) were mapped to the STRING database for prediction of DEGs-DEGs interactions. After removing the disconnected nodes and deleting undirected nodes, a total of 79 DEGs were tightly connected with confidence scores greater than 0.7. The predicted proteins were classified into seven large units, including units related to signal transduction, nitrate transport, nitrate metabolism, starch and sucrose metabolism, glycolysis, cell wall biosynthesis, and lignin biosynthesis metabolism processes. As shown in Figure 9 and Table S12, the DEGs related to signal transduction, starch and sucrose metabolism, and glycolysis were differentially regulated by nitrogen deficiency. As expected, DEGs related to nitrate metabolism and nitrate transport were tightly linked and differentially regulated in NO_3_^−^-deficient peanut roots. In the interconnected protein networks, nitrite reductase1 (NIR1) and nitrate reductase2 (NIA2) were interacted with NRT1.1 and NRT2.4, and all of them were among the downregulated proteins, while most of the DEGs associated with cell wall biosynthesis and lignin biosynthesis were upregulated by nitrogen deprivation. Furthermore, combined with previous studies, a putative schematic diagram of the inhibition of peanut roots by nitrogen deficiency was established (Figure 10).

### 2.11. Verification of RNA-Seq by qRT-PCR

To confirm the reliability of RNA-seq, five downregulated DEGs of two comparisons and five upregulated DEGs of HNR10 vs. LNR10 were randomly selected and examined by qRT-PCR. The result showed that the expression patterns of DEGs presented similar trends between RNA-seq and qRT-PCR (Table 3), suggesting that the RNA-seq data were reliable.

## 3. Discussion

### 3.1. Nitrate Acting as a Signal Regulating Root Morphology in Peanut Seedlings under Low-Nitrogen Condition

Previous studies have shown that plants intend to develop more exploratory root systems with longer lateral roots under nitrogen-deficient conditions [14,25]. However, the effect of nitrogen deficiency or low nitrogen on root branching may vary depending on the nitrogen nutrient status of the plants and the degree of deficiency to which the plants are subjected [26]. Remans et al. [27] found that the growth of lateral roots was inhibited in *Arabidopsis* in the absence of NO_3_^−^ or at low NO_3_^−^ concentration (0.05 mM). Gruber et al. [14] found that root length was significantly stimulated in *Arabidopsis* under moderate nitrogen (0.275–0.55 mM NO_3_^−^) deficiency; however, under severe nitrogen (0.11 mM NO_3_^−^) deficiency, root length was decreased, and the formation of the lateral root was almost completely absent. In this study, we found that root growth and development were significantly suppressed in the peanut seedlings cultivated in nutrient solution containing 0.1 mM NO_3_^−^ for 10 days (Figure 1, Table 1; [17]). Moreover, two genes encoding NRT1.1 and NRT1.2 were significantly upregulated at 10 days of low-nitrogen stress. The upregulated NRT1.1 may work as a NO_3_^−^ sensor, which may inhibit the growth of lateral roots by decreasing the accumulation of auxin in the lateral roots [13,27]. Several studies have also shown that the signaling action of NRT1.1 appears to be independent of its NO_3_^−^ transport activity [9,10,13,27,28]. The upregulated NRT1.2 may mediate cellular ABA uptake and promote the accumulation of ABA [29] and then inhibit the formation of lateral roots [30].

### 3.2. The Adaptive Mechanisms of Nitrate Transport and Assimilation under Low-Nitrate Condition

In higher plants, there are two types of NO_3_^−^ transporters, namely NRT1 and NRT2, which act as low-affinity nitrate transporters and high-affinity nitrate transporters, respectively [10]. Studies have shown that NRT1.1 has both a high-affinity absorption system and a low-affinity absorption system for NO_3_^−^ [31]. Further studies revealed that the affinity of NRT1.1 to NO_3_^−^ depends on its phosphorylation state [28], and its expression level is significantly induced by exogenous nitrate. Fan et al. [32] found that the mutation of the phosphorylation site of OsNRT1.1B protein is the main factor leading to the difference in nitrate uptake and transport between japonica and indica rice. Unlike NRT1.1, other reported NRT1s are NO_3_^−^ low-affinity transporters. AtNRT2.4 is a high-affinity NO_3_^−^ transporter, which was inhibited by NO_3_^−^ supply and played an important role in the absorption of NO_3_^−^ under low-nitrogen conditions [33]. CLC-a and CLC-b are NO_3_^−^/H^+^ reverse transport proteins involved in vacuolar nitrate storage. It was found that the expression of AtCLCa was significantly decreased under low-nitrogen stress, and the content of NO_3_^−^ in vacuoles of transgenic plants with silenced expression of AtCLC-a still significantly decreased under excessive NO_3_^−^ conditions [34]. In this study, a total of 13 DEG-encoded NO_3_^−^ transporters protein subunits (NRT1.1, NRT1.2, NRT2.4, WR3, and CLC-b) were significantly downregulated at 5 days of low-nitrogen stress and further downregulated with the extension of stress time. At the same time, nitrogen deficiency resulted in a significant reduction in the leaf nitrogen content, as indicated by the significantly decreased NBI value under 10 days of nitrogen deficiency, which may be due to the decreased NO_3_^−^ transporters protein.

After the NO_3_^−^ absorbed by the plant from the soil enters the plant body, it must undergo a series of nitrogen assimilation before it can be converted into organic nitrogen and then used by the plant. NR is the first step in the reduction of NO_3_^−^ and is also the rate-limiting step of nitrogen metabolism. Ferrario-Méry et al. [35] showed that NR activity level was positively correlated with its substrate concentration. In this study, the activity of NR was significantly decreased under both 5 days and 10 days of nitrogen deficiency condition, which may be induced by the decreased NO_3_^−^ content and the decreased NO_3_^−^ transporters protein. The activities of GS, GOGAT, and GDH were also significantly inhibited after 10 days of nitrogen deficiency treatment. These results are in accordance with a previous study [7], which indicated that low nitrate concentration decreased nitrogen assimilation. In addition, these results indicate that NR is more sensitive to low-nitrogen stress than GS, GOGAT, and GDH. In addition, the majority of genes encoding subunits of NR, NIR, GS, GOGAT, and GDH was downregulated during the nitrate assimilation pathway, which agrees with the nitrate assimilation enzyme activities.

### 3.3. The Mechanism of Plant Hormones Regulating Root Growth under Low-Nitrate Condition

Plant hormones and their signal transduction pathways were widely reported to participate in root development and the responses to nitrogen deficiency [36]. A number of DEGs involved in IAA, ABA, CTK, BR, JA, and SA synthesis or signal transduction were identified, indicating that these metabolic pathways might be related to nitrogen-deficient adaption in peanuts. It was first and most widely demonstrated that auxin is involved in root development, which is transported from the shoot to roots and promotes lateral root development [37]. The *aux1* [38] and *lax3* [39] mutants significantly decreased the number of lateral roots and repressed root elongation. In this study, one YUCCA protein-encoding gene (A.K71B7K) was downregulated under nitrogen stress, which is a key enzyme responsible for catalyzing the synthesis of auxin. At the same time, the genes (*A.R5YX1M* and *A.TK9VLP*) encoding the auxin influx carrier (AUX1) were also downregulated, which may result in a decrease in auxin content and, in turn, limit the occurrence and elongation of lateral roots [40]. In addition, one LAX3 protein-encoding gene (*A.LRNE2N*) was downregulated. Hence, we speculate that these DEGs were involved in the inhibition of peanut roots under nitrogen deficiency. Other research demonstrated that NRT1.1 was required to prevent auxin accumulation in lateral roots in *Arabidopsis* when external nitrate concentration was low [13]. Decreasing the expression of both NRT1.1 and AUX1 probably leads to auxin accumulation in the root apex and further inhibits root elongation.

It has been reported that NO_3_^−^ deficiency induces ABA accumulation, which inactivates its coreceptor ABA insensitive 2 (ABI2), a protein phosphatase 2C (PP2C) [41]. ABI2 can dephosphorylates the kinase CBL1-CIPK23 complex and increase the activity of CIPK23. Furthermore, CIPK23 could phosphorylate NRT1.1, which affects NO_3_^−^ absorption and signal transduction at low NO_3_^−^ concentrations (Figure 7) [28]. The phosphatase ABI2 mutant *abi2* in the ABA signaling pathway also exhibits a nitrate-related root growth differential phenotype, possibly by dephosphorylating CBL1/CIPK23-NRT1.1, which further suggests that ABA signaling is associated with NO_3_^−^ uptake and signal crossinteraction [42]. In this study, *A.R4CPVK* and *A.9ML3HZ* were upregulated by nitrogen deficiency, which encode the subunits of PP2C and CIPK23, respectively. These results are similar to the above reports, indicating that PP2C, CIPK23, and NRT1.1 genes are involved in peanut root development under low NO_3_^−^ concentrations.

### 3.4. The Relationship between Lignin Synthesis and Root Growth under Low-Nitrate Condition

The inhibition of root apical cell elongation is closely related to cell wall elasticity, which depends on the lignin content [43]. Lignin is mainly stored in the secondary cell wall and immobilizes the cell structure of plants [43]. Various biotic and abiotic stresses affected plant root elongation by regulating the expression of the genes associated with the lignin biosynthetic pathway [15]. It is widely reported that POD is an enzyme that catalyzes the last step of lignin biosynthesis (oxidative polymerization of lignin monomers) and plays a regulatory role in regulating cell wall stiffening [15]. During rapeseed root development, nitrogen deficiency decreased the expression of most PODs and suggested that lower POD activity promoted root elongation according to the loosening of roots [15]. In our study, only four POD protein-encoding genes were downregulated in the early stage under nitrogen deficiency. With the stress period increasing, more than half of POD protein-encoding DEGs and all DEGs encoding PAL, 4CL, HCT, F5H, OMT, and CAD were upregulated (Figure 8). The upregulation of key enzymes of lignin synthesis, such as PAL, 4CL, CAD, and POD, leads to the increase in lignin synthesis under low NO_3_^−^ stress, and the deposition of lignin closes the metabolic process of the plant cell wall, leads to cell wall lignification, and then inhibits root elongation [44].

### 3.5. The Response of TFs under Low-Nitrate Condition and Their Relationship with Root Growth

TFs play an important role in regulating gene expression in plant response to physiological changes, such as those from MYB, NAC, MADS, LOB, and GRAS families [45]. Of these TFs, several LOB family genes are closely involved with nitrogen uptake, assimilation, and root development. In *Arabidopsis*, LBD16 (lateral organ boundary domain) and LBD18 acted as positive regulators of lateral root number. For instance, the *lbd16*-*lbd18* dual mutants of *Arabidopsis* pronouncedly decreased the number of lateral roots [46]. In our study, two downregulated DEGs (*A.TBPN76* and *A.KW2NIT*) were homolog with LBD16-encoding genes of *Arabidopsis*, and the number of lateral roots was significantly decreased under nitrogen deficiency, indicating that these LBD16-encoding genes participated in the regulation of lateral root development. In addition, we also found that six downregulated genes encoded subunits of LBD37, 38, and 39. These results were contrary to those in *Arabidopsis*, which suggests that LBD genes may restrain many nitrate-responsive genes, including NRT, NR, and NIR protein-encoding genes [47].

### 3.6. The Adaptive Mechanisms of Carbon Metabolism under Low-Nitrate Condition

Carbon metabolism provides energy and carbon skeleton for nitrogen metabolism, and nitrogen metabolism provides important photosynthetic pigments and enzymes for carbon metabolism. The coordination of carbon and nitrogen metabolism significantly affects the efficiency of nitrogen metabolism of crops [48]. Under the condition of NO_3_^−^ deficiency, two sucrose synthase genes (SUS3 and SUS4), one starch synthase gene (At3g01180), and one starch branching enzyme gene (SBE2.2) were downregulated, while two β- fructofuranosidase genes (ATBFRUCT1 and BFRUCT4), two α- amylase genes (amy1 and amy2), and one α-glucan phosphorylase gene (PHS2) were upregulated. Both sucrose synthase and β-fructofuranosidase are key enzymes in plant sucrose metabolism, of which the former is responsible for catalyzing the decomposition of sucrose into uridine diphosphate (UDPG)-glucose and fructose, providing precursors for polysaccharide synthesis, and the latter can hydrolyze sucrose to produce fructose and glucose [49,50]. α-amylase and α-glucan phosphorylase catalyze the cleavage of α-1, 4 glycosidic bonds to decompose starch, while starch synthase and starch branching enzymes catalyze the formation of α-1, 4 glycosidic bonds and α-1, 6-glycosidic bonds, respectively, to synthesize starch [51]. There are also studies to show that an excess amount of nitrogen affected carbon metabolism [52]. The above results showed that low-nitrate stress affects carbon metabolism by inhibiting the decomposition of sucrose as well as accelerating the decomposition of starch. Glucose can generate pyruvate through glycolysis. On the one hand, pyruvate can generate acetyl coenzyme A under the catalysis of pyruvate dehydrogenase and then enter the tricarboxylic acid cycle under the action of citric acid synthase to completely oxidize and decompose to release energy. On the other hand, it can generate ethanol under the action of pyruvate decarboxylase and ethanol decarboxylase to release a small amount of energy. Under the condition of NO_3_^−^ deficiency, glycolysis pathway key enzymes 6-phosphofructokinase gene (PFK2), fructose-1, and 6-bisphosphatase gene (AT5G64380) and gene (LAT3 and AT1G54220) expression of encoding dihydrolipoic acid transacetylase, which was a component of the pyruvate dehydrogenase complex, were all upregulated, while the hexokinase (HXK1) gene was downregulated. At the same time, two gene (CSY3 and CSY2) expressions of encoding citrate synthase, which was a key enzyme in the tricarboxylic acid cycle, were all upregulated. The aldehyde dehydrogenase (ALDH3F1) gene involved in the gluconeogenesis pathway was upregulated, while the alcohol dehydrogenase (ADH1, AT1G32780) gene and two pyruvate decarboxylase genes (PDC2 and AT4G33070) were downregulated. In addition, the genes (G6PD1 and G6PD2) encoding 6-phosphoglucose dehydrogenase and genes (AT3G60750 and AT2G45290) encoding transketolase involved in the pentose phosphate pathway were all downregulated. This shows that the direct oxidation of glucose (pentose phosphate pathway) is inhibited. Therefore, the results of this experiment showed that the glucose generated from starch decomposition induced by low-nitrogen stress is converted into pyruvate through glycolysis and then enters the tricarboxylic acid cycle to completely oxidize and decompose to release energy to adapt to low-nitrate stress conditions.

In conclusions, we conducted a comparative analysis of morphology, nitrogen assimilation enzyme activities, and transcriptome between the control and nitrogen deficiency in peanut roots and found that nitrogen deficiency hampered peanut root development by suppressing the elongation and proliferation of roots, that the activities of NR, GS, GDH, and GOGAT were inhibited by nitrogen deficiency, and that a total of 293 (119 up- and 174 downregulated) and 2271 (1165 up- and 1106 downregulated) DEGs were induced after five and ten days of nitrogen deficiency, respectively. These DEGs in nitrate transportation and assimilation, the lignin biosynthetic pathway, plant hormone signal transduction, and transcription factors might be involved in peanut root development under nitrogen deficiency. In the future, to elucidate the function of these identified genes, more advanced genome tools, such as CRISPR/Cas genome editing [53,54], may be employed. Finally, a putative schematic diagram of the inhibition of peanut roots by nitrogen deficiency was proposed. Our data provide a better understanding of the mechanisms behind the root morphology responses and nitrogen metabolism to nitrogen deficiency in peanut roots and a theoretical basis for improving nitrogen use efficiency.

## 4. Materials and Methods

### 4.1. Plant Materials and Growth Conditions

Peanut (*Arachis hypogaea* L.) cultivar “Yuhua 37” was used to examine the influence of nitrogen deficiency on roots. “Yuhua 37” is an oleic-acid-rich pearl bean-type peanut variety selected by the Henan Academy of Agricultural Sciences, which is widely cultivated in the main peanut-producing area in China. Healthy and uniform seeds were selected and sterilized with 5% H_2_O_2_ for 5 min. Then, the seeds were washed with sterilized water to remove the residues of H_2_O_2_. Seed germination was performed with the roll germination paper method as described in a previous report [55].

One week after planting, emerged uniform seedlings were selected and transferred into plastic pots with 4.5 L of modified Hoagland nutrient solution, which contains the following components (pH 6.3 ± 0.1): MgSO_4_ (1 mM), KH_2_PO_4_ (0.5 mM), NaCl (2 mM), KCl (2 mM), EDTA-FeNa (0.1 mM), CuSO_4_ (0.2 μM), ZnSO_4_ (1 μM), H_3_BO_3_ (0.02 mM), (NH_4_)_6_Mo_7_O_24_ (0.005 μM), and MnSO_4_ (1 μM). To explore the influence of nitrogen deficiency, two nitrate concentrations were designed in this study. The low-nitrogen (LN) solution contained 0.05 mM Ca(NO_3_)_2_ and the high-nitrogen (HN) solution contained 2.5 mM Ca(NO_3_)_2_. Moreover, the ion concentration was complemented using 2.45 mM CaCl_2_ in LN. All seedlings were cultured under 14 h light at 30 ± 2 °C, followed by 10 h dark at 25 ± 2 °C. The root samples under HN treatment for five and ten days were defined as HNR05 and HNR10, respectively; the root samples under LN treatment for five and ten days were defined as LNR05 and LNR10, respectively.

### 4.2. Determination of Root Morphology and Dry Weight

After five and ten days of treatment, the roots were separated and scanned with the Epson Perfection V800 Photo scanner (Epsin America lnc., Long Beach, CA, USA). Then, the root morphology was obtained and a red bar 2 cm in length and 1 cm in height was used to represent the original size of root morphology. The total root length (TRL), total root surface area (TRSA), average root diameter (ARD), and total root volume (TRV) were calculated using WinRHZIO 2007 (Regent Instruments Inc., Québec, QC, Canada). The number of first-order lateral roots (NLR), i.e., lateral roots formed along the primary root, first-order lateral root district length (LRDL), and primary root length (PRL) were manually measured. After the measurement of root morphology, all roots were first put into an oven at 105 °C for 20 min and then transferred to a drying oven at 80 °C to constant weight. Finally, the root dry weight (RDW) was measured.

### 4.3. Determination of Nitrogen Balance Index (NBI)

Peanut plants with uniform growth were selected, the parietal leaves of the second pair of compound leaves were used as the measurement site, and the nitrogen balance index (NBI) of the leaves of each treatment was measured by a plant polyphenol-chlorophyll meter (Dualex Scientific+, Force-A, Ander-Loire, France).

### 4.4. Determination of Nitrate Assimilation Enzyme Activities

NR (nitrate reductase) activity was quantified according to the method presented by Jin et al. [56] with minor modifications. Briefly, for each sample, 0.1g of the root sample was powdered and transferred into a tube. Then, 5 mL of extracting solution (10 mM L-cysteine, 5 mM EDTA, and 25 mM potassium phosphate buffer (PH 8.7)) was added into each tube. Subsequently, 0.2 mL of the sample was transferred into each new tube, and 0.5 mL of 100 mM KNO_3_ and 0.3 mL of 2 mM NADH were added and then incubated with a water bath at 25 °C in the dark for 30 min. After incubation, 1 mL of 30% trichloroacetic acid solution, 2 mL of α-naphthylamine, and 2 mL of sulfanilamide were added. The mixture was then incubated at 25 °C for 15 min. Finally, 200 μL of the reaction was added to the microplate (Tecan Spark 10M, Corning Inc., New York, NY, USA). The absorbance at 540 nm was measured with a microplate reader. Enzyme activities of GS, GDH, and GOGAT were detected using the test kit (Keming, Suzhou, China) according to the manufacturer’s instructions.

### 4.5. RNA Isolation and cDNA Library Construction

Total RNAs were extracted from peanut roots using RNAprep Pure Plant Plus Kit (TIANGEN, Beijing, China). The integrity, purity, and concentrations of RNAs were examined by 1% agarose gels, the Agilent Bioanalyzer 2100 system (Agilent Technologies, Santa Clara, CA, USA), and Qubit^®^ RNA Assay Kit in Qubit^®^ 2.0 Flurometer (Life Technologies, West Sacramento, CA, USA), respectively. cDNA libraries were constructed using NEBNext^®^ Ultra^TM^ RNA Library Prep Kit for Illumina^®^ (NEB, Ipswich, MA, USA). Then, all libraries were sequenced on an Illumina Hi-Seq NOVA platform with 150 bp paired-end reads.

### 4.6. Genome Mapping and Differential Expression Analysis

Raw data were first cleaned by removing the noise reads, including adapter, reads containing a high content of unknown base (N), and low-quality reads. After filtering, all remaining data (clean data) were aligned with peanut reference genome (https://www.peanutbase.org/data/public/Arachis_hypogaea/Tifrunner.gnm2.ann1.4K0L/, accessed on 4 June 2021) with HISAT2 [57], which allowed one mismatch. All data of RNA-Seq were submitted to the National Center for Biotechnology Information (NCBI) database (accession number: PRJNA690197).

Gene expression was calculated by fragments per kilobase of transcript per million fragments (FPKM) [58]. Differential expression analysis was conducted using the DESeq2 method [59]. The differentially expressed genes (DEGs) were identified with *p* < 0.05 and |log2 (fold change)| ≥ 1.

### 4.7. Functional Annotation of DEGs

Gene Ontology (GO) (http://www.geneontology.org/, accessed on 5 October 2021) annotation of DEGs was conducted with GOseq R packages [60] based on the hypergeometric distribution. Metabolic pathway analysis of DEGs was performed with the Kyoto Encyclopedia of Genes and Genomes (KEGG) database (http://www.kegg.jp, accessed on 21 October 2021) and KOBAS software [61]. Since the function of most *Arabidopsis* genes has been illuminated, all protein sequences of DEGs were mapped to the *Arabidopsis* databases using STRING (https://string-db.org/, accessed on 15 March 2022) to further understand the gene function.

### 4.8. Reverse Transcription PCR and Quantitative Reverse-Transcription PCR (qRT-PCR)

cDNAs were synthesized using Quantscript RT kit (TIANGEN, Beijing, China). qRT-PCR was conducted with SuperReal PreMix Plus (TIANGEN, Beijing, China), and the gene relative expression was calculated with the 2^−ΔΔCt^ method [62]. Glyceraldehyde-3-phosphate dehydrogenase (*GAPDH*) was used as an internal reference [63]. For DEGs, Primer 5.0 software was used to design the primers. All primers used in this study are listed in Table S1. The reaction volume and conditions were operated following a previous report [17]. All qRT-PCRs were repeated three times and three biological replicates were run for each treatment.

### 4.9. Statistical Analysis

Data on morphology, the activities of nitrate assimilation enzymes, and gene expression were presented with mean values (*n* ≥ 3). The significant analysis was conducted using SPSS 22.0 software (SPSS Inc., Chicago, IL, USA) with Student’s *t*-test.

## Figures and Tables

**Table 1:** Effect of nitrogen deficiency on peanut root growth parameters.

Treatment	Processing Time (Day)	RDW (g/Plant)	TRL (cm/Plant)	TRSA (cm^2^/Plant)	TRV (cm^3^/Plant)	NLR (No./Plant)	LRD (cm/Plant)	PRL (cm/Plant)
HN	5	0.12 ± 0.03 ^a^	478.32 ± 41.36 ^a^	99.47 ± 13.36 ^a^	1.65 ± 0.30 ^a^	86 ± 9 ^a^	13.2 ± 0.51 ^a^	17.8 ± 1.06 ^a^
LN		0.11 ± 0.02 ^a^	456.44 ± 25.48 ^a^	92.88 ± 9.47 ^a^	1.51 ± 0.22 ^a^	83 ± 3 ^a^	13.0 ± 1.24 ^a^	16.6 ± 1.40 ^a^
HN	10	0.23 ± 0.02 ^a^	1315.93 ± 82.63 ^a^	236.63 ±12.08 ^a^	3.39 ± 0.15 ^a^	133 ± 9 ^a^	22.77 ± 0.38 ^a^	29.1 ± 2.57 ^a^
LN		0.17 ± 0.03 ^b^	915.42 ± 47.37 ^b^	161.11 ± 12.29 ^b^	2.26 ± 0.24 ^b^	110 ± 9 ^b^	16.0 ± 0.81 ^b^	19.1 ± 0.55 ^b^

Notes: Different letters indicate a significant difference at *p* < 0.05 between the high-nitrogen and low-nitrogen treatment on the same day. HN: high nitrogen; LN: low nitrogen; RDW: root dry weight; TRL: total root length; TRSA: total root surface area; TRV: total root volume; NLR: number of lateral roots; LRD: lateral root district length; PRL: primary root length.

**Table 2:** KEGG enrichment analysis of three groups’ DEGs with corrected *p*-value < 0.05.

Comparisons	KEGG Pathway	KO ID	DEGs	Corrected *p*-Value
HNR05 vs. LNR05	Starch and sucrose metabolism	ko00500	14	8.55 × 10^−3^
	Pentose and glucuronate interconversions	ko00040	13	2.98 × 10^−5^
	Phenylpropanoid biosynthesis	ko00940	11	4.67 × 10^−3^
	Glutathione metabolism	ko00480	9	1.65 × 10^−3^
	Nitrogen metabolism	ko00910	6	7.95 × 10^−4^
HNR10 vs. LNR10	Phenylpropanoid biosynthesis	ko00940	71	0
	Starch and sucrose metabolism	ko00500	58	1.27 × 10^−4^
	Glutathione metabolism	ko00480	36	1.24 × 10^−8^
	Cysteine and methionine metabolism	ko00270	22	4.31 × 10^−2^
	Nitrogen metabolism	ko00910	20	2.18 × 10^−8^
	Cyanoamino acid metabolism	ko00460	19	1.41 × 10^−2^
	Tyrosine metabolism	ko00350	18	1.46 × 10^−3^
	Phenylalanine metabolism	ko00360	16	1.07 × 10^−2^
	Isoquinoline alkaloid biosynthesis	ko00950	10	4.76 × 10^−2^
	Taurine and hypotaurine metabolism	ko00430	8	4.54 × 10^−2^

**Table 3:** qRT-PCR confirmation of DEGs in HNR05 vs. LNR05 and HNR10 vs. LNR10.

Gene ID	Protein Identify	HNR05 vs. LNR05		HNR10 vs. LNR10	
		RNA-Seq	qRT-PCR	RNA-Seq	qRT-PCR
*A.P1SHIC*	BTB/POZ and TAZ domain-containing protein	−5.41	−1.73	−7.26	−3.49
*A.X7K798*	ferredoxin--nitrite reductase	−2.35	−8.29	−7.27	−8.46
*A.VZZ6TG*	high-affinity nitrate transporter 2.4	−1.99	−5.92	−4.58	−6.10
*A.TY8HHB*	protein NRT1/ PTR FAMILY 6.3	−2.34	−5.16	−6.04	−5.75
*A.3895TY*	LOB domain-containing protein 38	−5.29	−5.52	−8.71	−6.75
*A.T73DV5*	cationic peroxidase 1-like	-	-	2.35	0.63
*A.5D9CQE*	anthranilate N-methyltransferase	-	-	2.18	1.26
*A.6255L4*	cellulose synthase A catalytic subunit 8	-	-	2.02	1.83
*A.R4CPVK*	probable protein phosphatase 2C	-	-	1.03	0.41
*A.QB6IYK*	cyclin-D3-1	-	-	1.15	1.17

Note: For the results of qRT-PCR, the negative values represent downregulated expression and the positive values represent upregulated expression.

## Data Availability

The data that support the findings of this study are available from the corresponding author upon reasonable request.

## Supplementary Materials

The following supporting information can be downloaded at: https://www.mdpi.com/article/10.3390/plants12040732/s1, Table S1: Primers used in this study; Table S2: Summary of data statistics of mRNA libraries; Table S3: Genes identified in this study; Table S4: Differentially expressed genes of HNR05 vs. LNR05; Table S5: Differentially expressed genes of HNR 10 vs. LNR 10; Table S6: Overlapped DEGs between HNR05 vs. LNR05 and HNR10 vs. LNR10 comparisons; Table S7: GO enrichment analysis of DEGs in both comparisons; Table S8: DEGs participated in nitrate transportation and assimilation; Table S9: DEGs participated in hormone signal transduction pathway; Table S10: DEGs participated in Lignin biosynthetic pathway; Table S11: Transcription factor prediction of DEGs; Table S12: Protein prediction of DEGs.
